# Nipah Virus Outbreaks in India: A Comprehensive Update

**DOI:** 10.7759/cureus.92420

**Published:** 2025-09-16

**Authors:** Shreya Veggalam, Jayashankar CA, Ojas Balaji, Mansi Thipani Madhu, Mir Hyder Hussain, Shraddha H, Manish GR, Koshy Sam, Snigdha Reddy, Gulam Saidunnisa Begum, Venkataramana Kandi

**Affiliations:** 1 Medicine, Prathima Institute of Medical Sciences, Karimnagar, IND; 2 Internal Medicine, Vydehi Institute of Medical Sciences and Research Centre, Bangalore, IND; 3 Medicine, Vydehi Institute of Medical Sciences and Research Centre, Bangalore, IND; 4 Biochemistry, College of Medicine and Health Sciences, National University, Sohar, OMN; 5 Clinical Microbiology, Prathima Institute of Medical Sciences, Karimnagar, IND

**Keywords:** henipavirus, india, kerala, nipah virus, outbreak, prevention, vaccine

## Abstract

The Nipah virus (NiV) poses a serious threat to public health. This is a result of its high pathogenicity and zoonotic transmission potential. Since its discovery, NiV has been connected to multiple outbreaks with a high death toll, underscoring the need for effective response and surveillance strategies. Transmission of the virus is strongly associated with its natural reservoirs, fruit bats, as habitat decline has increased the likelihood of zoonotic spillovers. For the development of targeted therapeutics, it is essential to ascertain the host's immune reaction to NiV. Although there are now no viable treatments or vaccines, there is hope because promising candidate vaccines are being researched. This article examines the pathophysiology, immune evasion strategies, transmission dynamics, taxonomical position, and structural features of the NiV, with an emphasis on the epidemiology of the virus in India. This study intends to improve understanding of complex viral risks and the effects of public health campaigns by gathering all available data on treatment and preventive techniques. This will ultimately lead to better epidemic planning and response tactics.

## Introduction and background

The 21st century is witnessing several novel and re-emerging viral diseases caused by the severe acute respiratory syndrome coronavirus (SARS-CoV), the Middle East respiratory syndrome coronavirus (MERS-CoV), the Ebola virus (EBOV), the Mpox virus (MPXV), and SARS-CoV-2. The Nipah virus (NiV) is one of several new bat-borne zoonotic viruses that pose an imminent public health threat [1]. NiV naturally inhabits the fruit bats belonging to the *Pteropodidae *family, also known as flying foxes. Humans can contract NiV from animals (like pigs or bats), food contaminated with bat body fluids (saliva, urine), and feces, or an infected person. The clinical spectrum is diverse, ranging from asymptomatic or mild febrile illness to acute respiratory disease and severe, often fatal encephalitis [2, 3]. Numerous domestic animal species, including pigs, horses, dogs, and cats, are susceptible to NiV infection. In pigs, illness results in respiratory and sometimes neurological symptoms. Neither a human nor an animal vaccine nor specific antivirals are currently available, and treatment remains limited to supportive care. This therapeutic gap underscores the urgent need for the development of appropriate preventive strategies against the NiV [4].

NiV was first discovered during an outbreak in Malaysia in 1998-1999, following which it has been connected to numerous outbreaks around Asia, particularly in Bangladesh and India, that have caused a considerable amount of mortality with consistently high case fatality rates (CFRs). The two genetically distinct strains of NiV, the Malaysian strain (NiV-M) and the Bangladeshi strain (NiV-B), possess an enclosed negative-stranded ribonucleic acid (RNA) genome. The NiV-B has been linked more strongly to human-to-human transmission and higher pathogenicity compared to the NiV-M variant. The interactions between human activity and fruit bats, a species of Pteropus, are intimately linked to the virus's development and transmission [5].

In India, outbreaks have been documented in 2001, 2007, 2018, 2019, 2021, and 2023, highlighting its potential for re-emergence and the significance of efficient surveillance, preventive, and response measures. Because of its CFR and the risk of spreading through various species and human-to-human transmission, researchers see NiV infection as a complex public health challenge. A recent study highlighted a mortality rate of 73.9% among NiV-infected patients, underscoring its serious impact and the significant public health threat it presents during potential outbreaks [5].

The pathophysiology, immunological evasion tactics, transmission dynamics, and structural characteristics of the NiV are examined in this article, with a focus on the virus's epidemiology in India. By compiling all current information regarding treatment and prevention strategies, this study aims to enhance knowledge of complex viral threats and the impact of public health campaigns, ultimately resulting in improved outbreak preparedness and response strategies.

## Review

NiV taxonomy and characteristics

**Figure 1:**
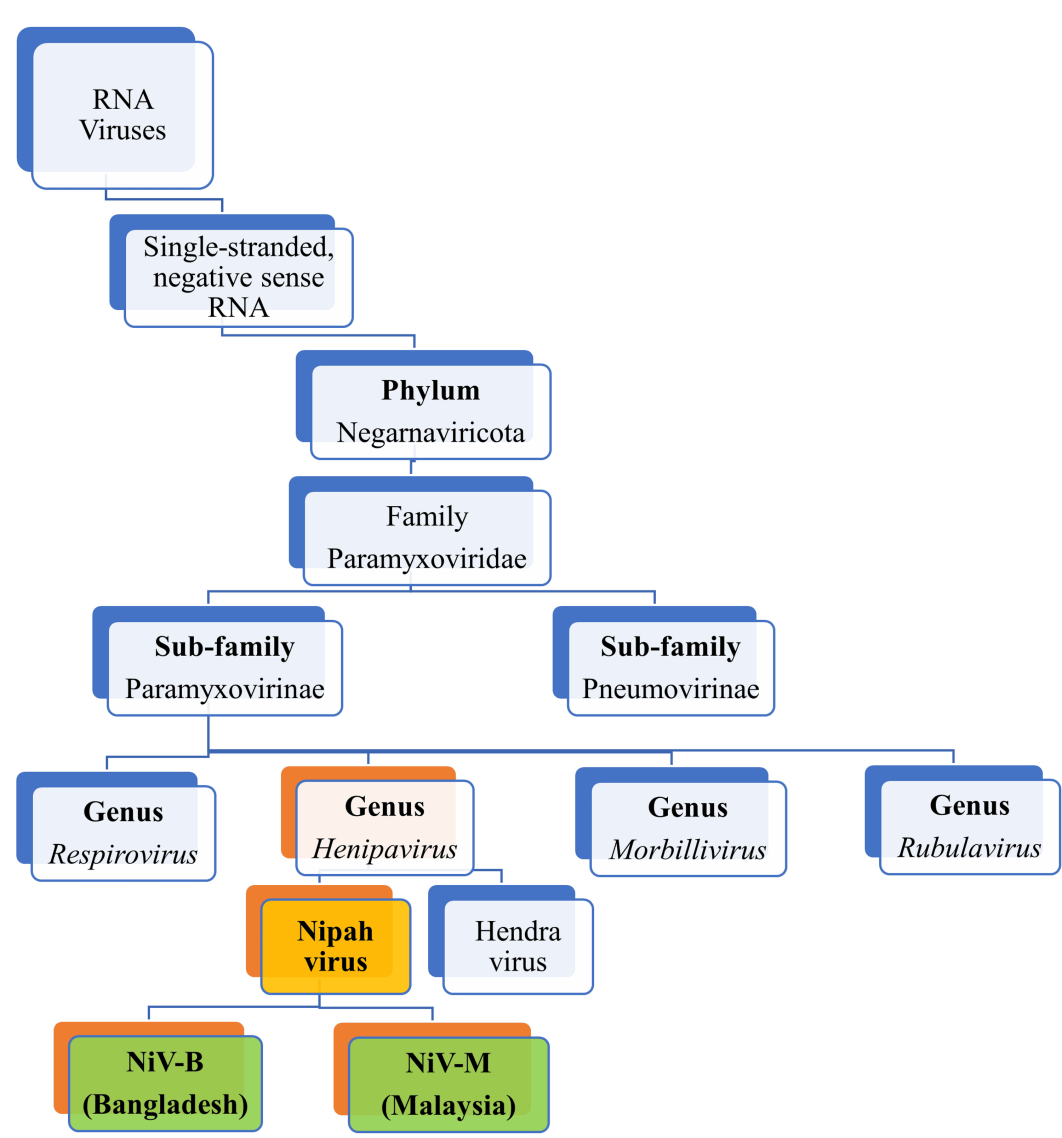
Taxonomy and types of Nipah virus Image credit: Venkataramana Kandi This image has been synthesized from reference [6]. RNA: ribonucleic acid; NiV-B: Nipah virus-Bangladeshi strain; NiV-M: Nipah virus-Malaysian strain

The virus was first identified in Sungai Nipah village located in Malaysia, which is where the term "Nipah" comes from [6]. The genus Henipavirus, which includes the NiV, is a member of the Paramyxoviridae family (Figure 1).

NiV is a pleomorphic, enveloped virus that ranges in diameter from 120 to 150 nm. NiV demonstrates a helical nucleocapsid covered by a single layer of surface projections. NiV comprises a negative-stranded RNA genome, which is unsegmented and measures approximately 18 kilobases (kb) in length. The NiV genome codes for structural proteins including nucleoprotein (N), phosphoprotein (P), matrix protein (M), fusion (F), attachment (G) glycoproteins (GPs), and RNA polymerase (L). The F and G GPs make up the virus's bilipid outer coat. The viral genome also codes for ribonucleoprotein produced by the nucleocapsid, the phosphoproteins, and the polymerase proteins, along with the F and G GPs, that facilitate host cell entry [7, 8]. Additionally, the NiV genome codes for three accessory or non-structural (NS) proteins, including the C, V, and W proteins [9-11]. The NS proteins work as virulence factors by interfering with the host's immune system and taking over cellular processes to facilitate infection. Attachment GPs bind to host cell ephrin B2/B3 receptors, facilitating viral fusion with host cell membranes. The viral entry receptors are primarily expressed on neurons and endothelial cells. This explains the vasculotropic and neurotropic properties of NiV [12-14].

The clinical presentation, pathogenicity, and mortality of NiV infection vary among different populations for the two genetically distinct strains, including NiV-M and NiV-B. Identifying the specific NiV strain that caused the outbreak is crucial for characterizing transmission dynamics and forecasting public health emergencies. Retrospective identification of the virus using stored samples from patients during the outbreak helped identify the first strain, the NiV-M, during the first-ever outbreak in Malaysia in 1988. Since pigs were linked to it, it was believed that they were the source of the zoonotic transmission. The second strain, which is linked to fruit bats, was the NiV-B [15]. In addition to zoonotic spillover, the NiV-B has been shown to exhibit human-to-human transmission. This could be the reason for the frequent outbreaks in Bangladesh, Malaysia, and India. Additionally, NiV-B is determined to be more virulent than NiV-M [16, 17].

Reservoirs and transmission dynamics

Fruit bats belonging to the *Pteropus *genus are the main natural reservoir of the NiV. Geographically, South Asian countries are home to a large number of fruit bats, which are also the primary cause of the region's frequent outbreaks. These bats remain asymptomatic and carry the virus. Fruits and palm sap that people or animals may consume become contaminated by the virus, which is excreted through bat urine, feces, and saliva. When people eat tainted date palm tree products, they become infected and exhibit a variety of symptoms, ranging from mild headaches to severe respiratory distress and, in the majority of cases, death [18, 19]. According to recent research, very harmful viruses such as the rabies virus and related lyssaviruses, NiV and Hendra virus (HeV), as well as SARS-CoV-like viruses, are found in bats. Neutralizing antibodies (nABs) against NiV were detected in fruit bats through serological surveillance, indicating a close relationship between the virus and the bats [20].

In Bangladesh, it was revealed that humans can contract NiV from dead people. When someone dies, the body is bathed before being buried as part of local traditions and customs. The ritual participants contracted the virus from the corpse, and it was discovered that the respiratory and vaginal secretions of the deceased, to which they were exposed throughout the ritual, may have been the means of transmission. There was proof of human-to-human transmission during the epidemics in Bangladesh and India. The primary targets of this route of infection are the close family members and medical professionals who work closely with the infected individuals [21].

Consuming raw date palm sap, which is likely contaminated by bat secretions like saliva, and being exposed to fruit bats were directly linked to the latest outbreaks in India. Research shows that the oropharyngeal secretions of cats and pigs can spread the disease asymptomatically. Pigs serve as intermediate hosts, accelerating the spread of the disease and leading to major epidemics in agricultural occupations centered around pig farms [22, 23]. The diverse range of hosts highlights the broad extent of zoonotic transmission. This confirms the NiV's adaptability to multiple species and potential for zoonotic spillovers. In most cases, the animals do not exhibit any symptoms. However, the virus severely affects humans who come into direct or indirect contact with infected animals, such as horses, dogs, and pigs [24].

In artificial date palm sap, the NiV can endure for at least seven days, but it can only survive for three days in fruit juices or mangoes. Since it can live on surfaces, the fomite mechanism of transmission should be considered. At temperatures as high as 70°C, NiV can persist for an hour in the environment and has an 18-hour half-life in bat urine [25, 26]. Because of its high fatality rates and potential for zoonotic and human-to-human transmission, NiV is regarded by the World Health Organization (WHO) as a priority infection [2]. Since there is currently no particular cure for the virus, both prognosis and treatment are extremely challenging.

Clinical presentation

Moderate- to high-grade fever, altered sensorium, shortness of breath, generalized weakness, coughing, and symptoms of diffuse vasculitis and atypical pneumonia begin the NiV presentation, followed by an abrupt progression to encephalitis. The NiV infection incubation period is predicted to be 7-11 days. Few or no asymptomatic cases have been recorded to date. Because the virus is both neurotropic and vasculotropic, it has a significant impact on the respiratory and neurological systems. Acute respiratory distress syndrome (ARDS), multiorgan failure, and death are the results of the ensuing decline [27, 28].

There are extremely few survivors of the disease, and its CFR is extremely high. Long-term neurological and functional effects were the main focus of studies conducted on the survivors of the Bangladesh pandemic. Survivors of NiV-induced acute encephalitis will unavoidably experience functional morbidity and lasting neurological impairments for the rest of their lives. The process by which the virus enters a latent condition within the body and reactivates is unknown. The remaining symptoms align with facial paralysis, focal weakness, cervical dystonia, ocular motor palsy, and static encephalopathy. Cerebral atrophy, multifocal hyperintensities, and confluent cortical and subcortical signal alterations are examples of MRI abnormalities. For vulnerable people, the infection might be lethal within a few days [29].

Fever, headache, myalgia, vomiting, and altered sensorium, which could range from confusion to coma, were among the symptoms noticed in patients during the 2001 NiV outbreak. Of the patients, 43% experienced convulsions or involuntary movements, and 51% experienced respiratory symptoms such as tachypnea and acute respiratory distress. No signs of stiff neck or injury to the cranial nerve were found [30].

The symptoms of the 2018 NiV outbreak in Kerala included encephalitis, fever, vomiting, and agitation. Complications included viral bronchopneumonia and ARDS in 36.84% of patients. Myocarditis, ARDS, and bronchopneumonia accompany encephalitis in 26.31% of patients. Nineteen of the 17 serum samples that underwent polymerase chain reaction (PCR) and serological testing had NiV-specific immunoglobulin (Ig) M and IgG. IgM was negative in one sample, whereas IgG was positive [31, 32].

NiV RNA was detected by reverse transcription-PCR (RT-PCR) in four urine samples from patients with positive antibodies and one sample from a patient without positive antibodies. The M gene RNA was found in three out of the five samples. No matter if the case was suspected or probable, a confirmed NiV case was defined as a blood sample that tested positive for anti-NiV Ig M by enzyme-linked immunosorbent assay (ELISA) or a positive NiV RNA result from real-time RT-PCR of any bodily fluid [31, 32]. Magnetic resonance imaging (MRI) of NiV-induced acute encephalitis syndrome (AES) showed multiple bilateral mild abnormalities in deep white matter and subcortical areas. In certain instances, the corpus callosum, cortex, and brainstem were also impacted [31, 33].

A weighted MRI also revealed broad acute cytotoxic edema, which suggested the presence of a viral infection. Diffusion-weighted MRI showed that the lesions in the patients either vanished or grew less visible over time. Repeated MRIs revealed novel transient hyperintensities in the cerebral cortex that mirrored laminar cortical necrosis and provided insight into the mechanism of NiV infection [34, 35].

Pathogenesis

The virus enters the body through respiratory droplets, aerosols, and oral intake/ingestion. Additionally, it can spread by direct contact with broken skin and mucous membranes. The respiratory epithelium is where the virus particles settle. In this case, the virus's glycoproteins bind to ephrin B2/B3 receptors of the respiratory epithelium. Through further procedures involving the viruses' fusion and entry proteins, the viral genome enters the cell. Replication of the virus occurs in the respiratory epithelium and spreads through the circulation to the body's organs, particularly the endothelial cells in the brain, lungs, kidneys, and gastrointestinal system. Additionally, it has been demonstrated that the virus penetrates the central nervous system (CNS) through the olfactory nerve [36-38].

Retrograde transmission through olfactory neurons and hematogenous dispersion through the choroid plexus or blood vessels in the brain are two ways that NiV can enter the CNS [36]. The disruption of the blood-brain barrier caused by CNS infection leads to the expression of tumor necrosis factor alpha (TNF-α) and interleukin (IL)-1 beta (IL-1β), both of which are associated with neurological symptoms [37, 38]. Since NiV infection involves the epithelial cells of the bronchioles, the virus most likely entered the body through the nose [39]. IL-1α, IL-6, IL-8, granulocyte-colony stimulating factor, and C-X-C motif chemokine 10 are among the inflammatory mediators that rise in response to an infection of the airway epithelium [40].

**Figure 2:**
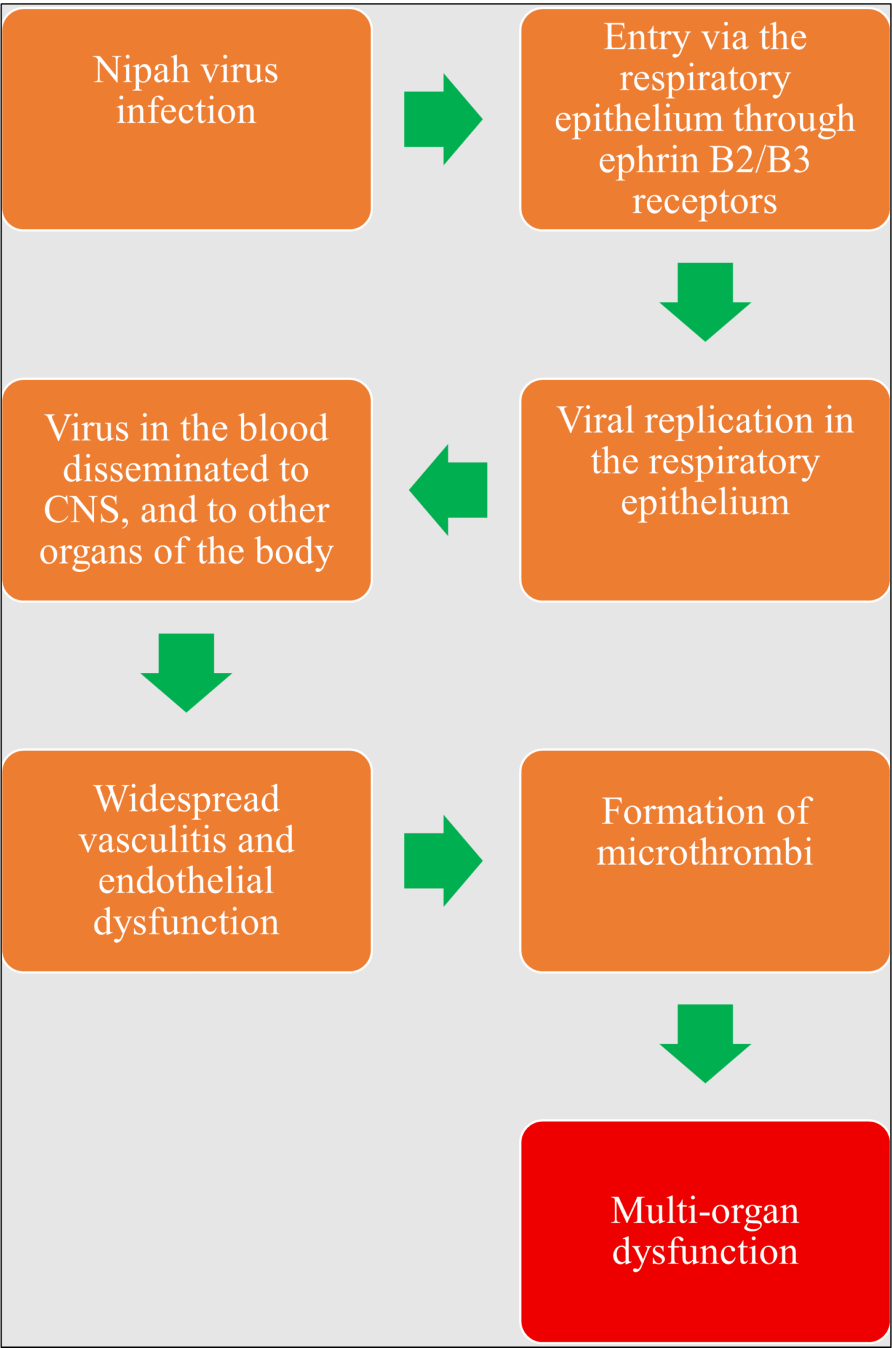
The pathogenesis of Nipah virus Image credit: Shreya Veggalam This image has been synthesized from references [36-42]. CNS: central nervous system

An infection of the respiratory epithelium triggers an inflammatory response that draws immune cells and causes symptoms similar to ARDS. As the disease progresses, the virus travels from the respiratory epithelium to the endothelial cells of the lungs, eventually entering the circulation. It can attach itself to host leukocytes or circulate freely throughout the body [37, 40]. The NS proteins, such as proteins V and C, are upregulated as a result of viral replication (Figure 2) [41, 42].

Immune response

When exposed to the NiV, the immune system becomes activated. Nevertheless, the host is weakened, and significant mortality rates result from the viral antagonistic processes that attempt to elude the immune response. The NiV effectively suppresses the production of antiviral cytokines in the early phases of infection, promoting inflammation and vascular permeability, both of which enable the virus's spread. Lethal encephalitis may result from the virus's ability to evade immune responses for extended periods, which is linked to its persistence in the brain [43]. The virus modifies genes related to immune defense, neurogenesis, and the blood-brain barrier. Additionally, the genome contains microRNAs (miRNAs) that inhibit these host genes, which increases the virus's pathogenicity and spread [44].

Thymus (T)-cell responses may be hampered by the NiV's ability to induce widespread necrosis of lymphoid tissue and immune cell death. The innate or adaptive immune responses alone cannot stop viral propagation. The inability of dendritic cells to efficiently eliminate the virus leads to an insufficient immunological response. Additionally, by preventing immune cells from producing major histocompatibility complex-1 (MHC-I), the NiV reduces adaptive immune responses and impedes antigen presentation, allowing the virus to persist and spread to other organs. NiV causes a hyperinflammatory reaction that results in vasculitis and severe endothelial impairment [39, 45, 46].

From asymptomatic to catastrophic sickness and death, the outcome of NiV infection depends entirely on the host's resistance and the viral ability to infect the host [47]. It may be possible to identify potential pharmaceutical targets if the intricate immune system mechanisms against the virus are properly understood. As a result, future drug development for treatment may be made easier. For this path to be successful, more human and animal research and studies are needed.

Laboratory diagnosis

NiV infection in a patient’s blood sample can be indirectly detected using anti-NiV IgM and IgG detection methods, such as virus neutralization tests and ELISA, including the direct ELISA, indirect ELISA, and sandwich ELISA. Direct detection methods, including whole-genome sequencing (WGS) or next-generation sequencing (NGS), virus culture, immunohistochemistry, immunofluorescence, and nucleic acid amplification, are employed to confirm the diagnosis using patients’ specimens such as blood, throat swabs, nasal swabs, urine, and cerebrospinal fluid (CSF). When diagnosing patients, a quantitative RT-PCR (qRT-PCR) is a preferred technique to identify viral nucleic acid from urine, blood, nasal swabs, and CSF specimens [5, 9-11, 48].

Other techniques, including real-time RT-PCR and nested RT-PCR, confirm the diagnosis. RNA templates are amplified using nested RT-PCR. This highly sensitive method converts RNA into complementary deoxyribonucleic acid (cDNA) before amplifying it using nested primers, improving the identification of low-abundance targets. Besides, the pathogen nucleic acid is detected in patient samples using real-time RT-PCR, which employs DNA probes [49].

The TaqMan real-time PCR, SYBR Green assay, real-time RT-loop-mediated isothermal amplification (rt-RT-LAMP), and rapid molecular diagnostic tests are alternative methods to diagnose NiV infection. TaqMan real-time PCR uses a TaqMan probe to quantify the viral burden. SYBR Green is also a quantitative PCR that employs SYBR Green I fluorescent dye (N’, N’-dimethyl-N-[4-[(E)-(3-methyl-1,3-benzothiazol-2-ylidene)methyl]-1-phenylquinolin-1-ium-2-yl]-N-propylpropane-1,3-diamine) that can bind to DNA. Previous reports found no evidence of cross-reactivity of NiV diagnostics with HeV, respiratory syncytial virus (RSV), EBOV, or Lassa virus (LASV) [9, 48, 49].

Low- and middle-income countries (LMICs) with resource and financial limitations can benefit from rapid tests based on isothermal amplification techniques like recombinase polymerase amplification (RPA) (TwistDX, Cambridge, United Kingdom: TwistAmp® nfo kit and the TwistAmp® exo kit) and recombinase-aided amplification (RAA) (Qitian, Jiangsu, China). These tests are low-cost, easy to do, and can be used as point-of-care (POC) tests [50].

Outbreak of NiV in India

A few notable sporadic outbreaks have occurred in India and caused high fatality rates. Two outbreaks occurred in West Bengal in 2001 and 2007, and the consequent reports from Kerala in 2018, 2021, and 2023 [51]. The reservoir for NiV is the fruit bats, which reside near human residences. The virus does not affect the bats, which remain asymptomatic. Therefore, bats are asymptomatic vectors that spread the virus through their body fluids and excreta [4]. Human-to-human transmission is also reported due to close contact with infected individuals. Studies on healthcare workers who directly cared for encephalitis patients revealed very few of them were found to be positive for antibodies against the NiV, undermining the potential for nosocomial transmission [52]. Another major risk factor in India is the consumption of raw date palm sap contaminated by bat body fluids and excreta, like saliva, urine, and feces. Deforestation leads to the destruction of animal habitats, predisposing to increased human-to-animal interaction [31, 33].

The first outbreak in the Siliguri district of West Bengal in 2001 displayed feverish symptoms and altered sensorium among infected patients. The transmission during this outbreak was mainly via human interactions and the zoonotic spread, which led to the high case fatality rates. A total of 66 probable cases and 45 deaths were reported. The tests conducted on the stored serum of the patients after the outbreak revealed evidence of NiV. The major cause of the outbreak was found to be human-to-human transmission and nosocomial infection. According to the observations and patient history, the affected patients were adults, and pigs were not involved in the transmission of the disease [31, 33].

The next outbreak occurred in Belechuapara village, Nadia district, West Bengal, in the year 2007. A total of five patients were severely affected, and all of them succumbed to death within a week of the infection. This indicates a 100% CFR of the infection. The subsequent outbreaks, which occurred in Kerala in 2018, 2021, and 2023, were found to have originated from fruit bats. The primary risk factor identified was the consumption of raw date palm sap and contact with the infected patients [6, 31, 33].

A total of 17 deaths have been reported due to the infection in May 2018 in Kerala out of 18 confirmed cases, which represents a high CFR (94.44%). At first, a family of three was severely affected and died, along with the healthcare worker who was directly involved in the family's care. Laboratory tests confirmed the presence of NiV using RT-PCR and ELISA. There was high mortality and morbidity reported during these outbreaks. The WHO stepped up and supported the Indian and Kerala state governments' initiatives in the prevention and control of the disease during the three outbreaks (Table 1) [53].

**Table 1:** NiV outbreaks in India This table has been created by the authors. This table has been synthesized from references [31, 32, 54-61]. CFR: case fatality rate; NiV: nipah virus; rt-RT-PCR: real-time reverse transcription polymerase chain reaction; ELISA: enzyme linked immunosorbent assay; ARDS: acute respiratory distress syndrome; AES: acute encephalitis syndrome; IgG: immunoglobulin gamma; IgM: immunoglobulin mu; CSF: cerebrospinal fluid

Year	Location	Cases (n)	Deaths (n)	Case fatality rate (CFR) (n%)	Source of infection	Key features of the outbreak	Diagnostic methods used	Reference
July 2025	Malappuram district, Kerala	1	1	100%	The index case: an adolescent girl	Only the index case succumbed to death. 425 contacts of the primary case were identified, and widespread surveillance resulted in successful containment	rt- RT-PCR and ELISA for IgM and IgG antibodies to NiV	[54]
September 2024	Malappuram district, Kerala	1	1	100%	The index case: a 24-year-old male developed AES	Only the index case succumbed to death, and the infection was successfully contained	rt-RT-PCR and ELISA for IgM and IgG antibodies to NiV	[55]
July 2024	Malappuram district, Kerala	1	1	100%	The index case: a 14-year-old boy developed AES	Only the index case succumbed to death, and the infection was successfully contained	rt-RT-PCR and ELISA for IgM and IgG antibodies to NiV	[56]
2023	Kozhikode, Kerala	6	2	33.3%	The infected bats and human-to-human transmission	Cases had a mixed presentation of ARDS and AES	rt-RT-PCR and ELISA for IgM and IgG antibodies to NiV	[57]
2021	Kozhikode, Kerala	1	1	100%	The index case: a 12-year-old boy developed AES	Only the index case succumbed to death, and the infection was successfully contained	rt-RT-PCR and ELISA for anti-NiV bat IgG	[58]
2019	Ernakulam, Kerala state	1	1	100%	The index case: a 21-year-old male patient	Only the index case succumbed to death, and the infection was successfully contained	rt-RT-PCR and ELISA for anti-NiV bat IgG	[59]
2018	Kozhikode, Kerala	23	21	91%	Evidence of spread through touching, feeding, or nursing, and exposure to respiratory droplets from a NiV-infected person	Median incubation period was 9.5 days (range, 6-14 days). Of the 23 cases, 20 (87%) had respiratory symptoms	rt-RT-PCR analysis of throat swabs, blood, urine, and CSF specimens to detect NiV.	[32]
2007	Belechuapara village, Nadia district, West Bengal	5	5	100%	The index case: a 35-year-old male patient; consumption of raw date palm sap was identified as the source of infection. Hundreds of bats were observed hanging from the trees around his residence, which strongly suggested an association with the infection and the possibility of contamination of sap with bat excreta or secretions. There was evidence of possible human-to-human transmission	Affected individuals presented with AES, characterized by symptoms such as fever, headache, altered mental status, and seizures	Nested RT-PCR and ELISA for IgM and IgG antibodies to NiV	[60]
2001	Siliguri, West Bengal	66	45	68.18%	The major cause of the outbreak was found to be human-to-human transmission and nosocomial (hospital-based/healthcare-associated) transmission	Patients presented with symptoms of AES, including fever, headache, altered mental status, and seizures	Samples from stored patient specimens were tested using RT-PCR and ELISA.	[31, 61]

The Kerala state situation in terms of the NiV

Kerala has emerged as a recurrent hotspot for the NiV outbreaks in India. The first outbreak occurred in the year 2018 in the Kozhikode district [32]. The subsequent outbreaks were reported in the adjacent districts. The extensive flora and fauna, with huge populations of fruit bats, result in ecological vulnerability. Human encroachment into animal habitats also has a significant impact on the spread of zoonotic diseases.

**Figure 3:**
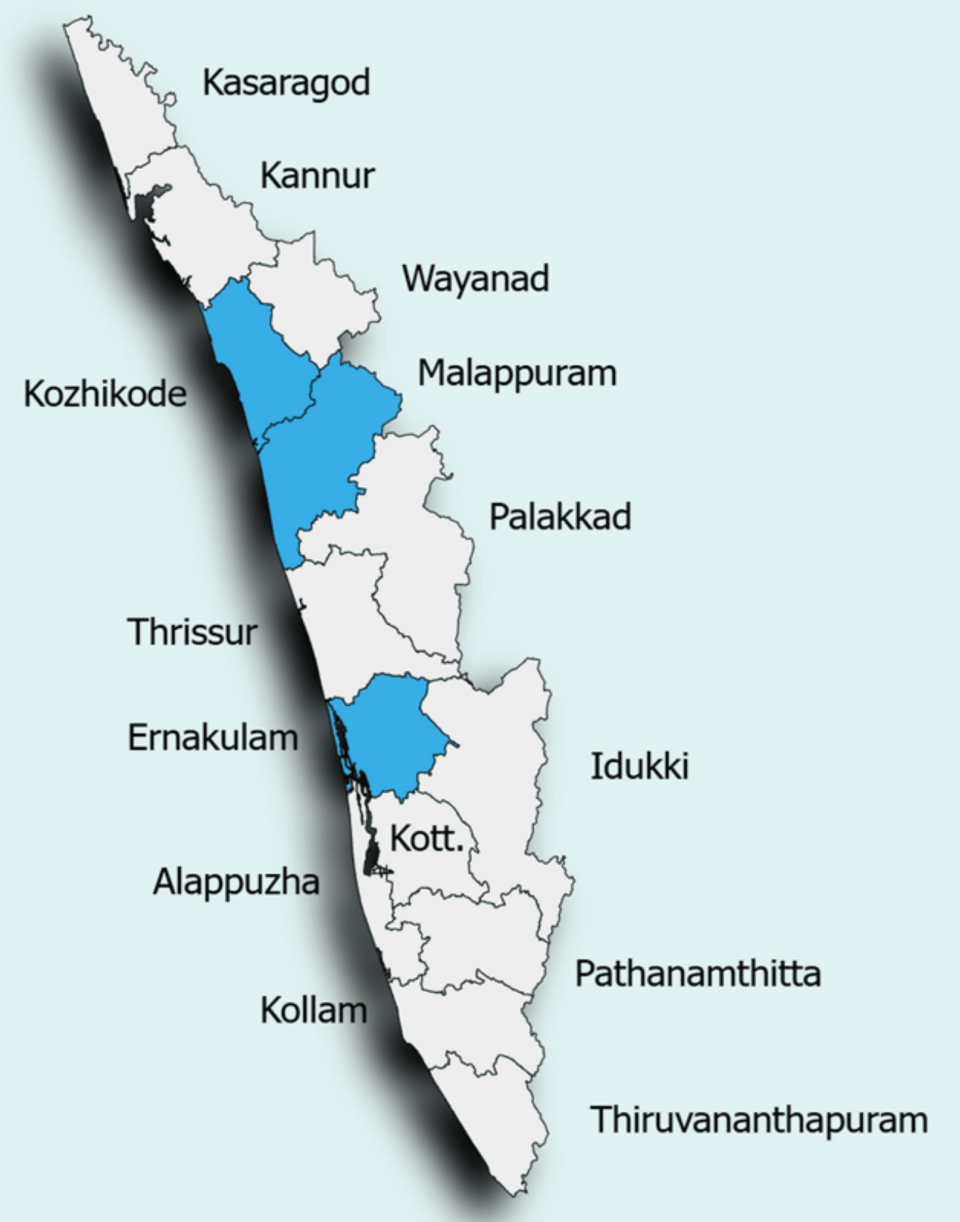
The Kerala state map highlighting (blue) the locations from where the Nipah virus infections were reported Image credit: Shreya Veggalam. The author has created the map on https://www.mapchart.net/. The map is licensed under a Creative Commons Attribution-ShareAlike 4.0 International License. This image has been synthesized from references [32, 54-59]. Blue highlighted districts: Kozhikode, Mallapuram, Ernakulam Kott.: Kottayam

Through efficient surveillance and healthcare activities, the Kerala state government contained the illness and prevented the subsequent epidemics from spreading to a wide population. In contrast to other common communicable viruses, NiV, which has a high case fatality ratio, places significant pressure on the administration, as it disrupts families and has a substantial impact on the economy, making it urgent to halt its spread and mortality. Along with public education, the state health department implemented stringent isolation protocols, community involvement tactics, and thorough contact tracing. Additionally, they adopted the One Health strategy, which involves preventing the transmission of zoonotic diseases and monitoring the health of both humans and animals [51, 54-59]. The state map of Kerala illustrates the districts that reported confirmed human cases of the NiV outbreaks, as shown in Figure 3.

A global view highlighting the NiV situation in South Asia

**Figure 4:**
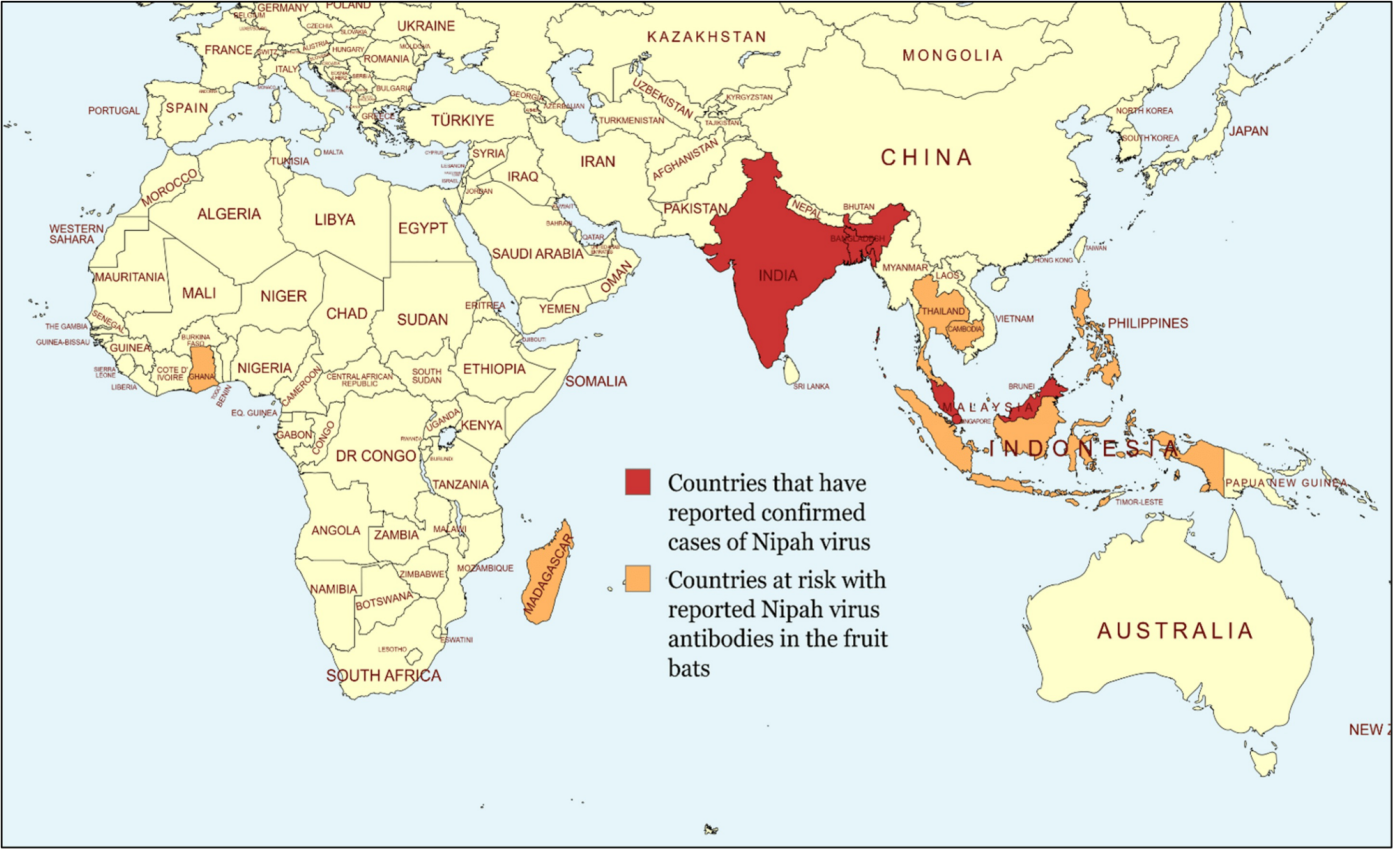
World map highlighting at-risk (orange) regions and the locations from where the Nipah virus infections (red) were reported Image credit: Shreya Veggalam. The author has created the map on https://www.mapchart.net/. The map is licensed under a Creative Commons Attribution-ShareAlike 4.0 International License. This image has been synthesized from references [2, 6]. Red: India, Bangladesh, Malaysia, Singapore; Orange: Thailand, Cambodia, Indonesia, Borneo Islands, Philippines, New Guinea, Ghana, Madagascar

The map indicates the countries that have reported confirmed cases of NiV, including India, Malaysia, Bangladesh, and Singapore. It also shows at-risk countries, including the Philippines, Thailand, Indonesia, and Madagascar, which reported anti-NiV antibodies in fruit bats. The geographic clustering of NiV outbreaks around the Bay of Bengal and the Indian Ocean reflects the recurrent hotspot countries and the possibility of viral transmission through shared fauna in neighboring regions (Figure 4) [2].

Treatment and prevention

Since there is no specific, standardized treatment for NiV, the management is supportive and offers palliative care to the patients. Professional supportive care, which includes symptom management, relaxation, and hydration, remains the sole treatment option for NiV infections. Monoclonal antibodies (mAbs), nucleotide or nucleoside analogues, antiviral agents, and fusion-inhibitory peptides are explored as therapeutic approaches.

mAbs

An example of an immunotherapeutic mAb-based intervention is m102.4. It targets the interaction between the host epithelium's ephrin B2/B3 receptors and the viral G GP, which was the focus of phase 1 clinical studies. Although this has not yet been proven in human models, the medication has been tested in animal models and produced positive outcomes. Since then, it has been used empathetically for NiV patients, and initial evaluations suggest that it holds promise [62]. By binding to quaternary F GP epitopes, humanized antibodies 5B3 and humanized 5B3.1 (h5B3.1), which are undergoing in vitro testing, can also block viral penetration and virus-host cell membrane adhesion. These have the potential to be used therapeutically in the future [63]. The neutralizing antibody (nAB) nAH1.3 is similar to m102.4. Both of these block the virus from entering the host cell via the same mechanism. They can work together to improve the therapeutic outcome [64].

Nucleotide and Nucleoside Analogs

Remdesivir is an RNA polymerase inhibitor that has demonstrated efficacy in reducing NiV infection in preclinical studies when administered to exposed nonhuman primates. In the test animals, its therapeutic efficacy was well-established. As an adjuvant therapy for immunotherapies such as m102.4 mAbs, it is now under investigation [65,66]. Another RNA polymerase inhibitor, favipiravir, helped lower virus titers in the Vero cell culture in a dose-dependent way. It has been demonstrated that favipiravir lowers mortality in hamster experimental models. In addition to mABs, the medication can be utilized to treat henipaviral infections for a synergistic effect [67]. The drug ribavirin was used during the 1999 NiV outbreak in Malaysia and given to a small number of patients. However, it is unclear how well it works to treat NiV. When treating the identified patients, stringent infection control procedures should be followed to minimize mortality and restrict human-to-human transmission [68].

Fusion-Inhibitory Peptides

By altering the confirmation of the F protein, this class of cholesterol-tagged peptides is utilized to restrict the amount of virus that may enter the host cell. According to the results of recent trials conducted on golden hamsters, their antiviral efficiency has dramatically increased, suggesting that they could be used as therapeutic agents in the future [69].

The modalities listed above are currently being investigated, and the safety, efficacy, and potential clinical use of these treatments in humans require further investigation.

Infection prevention and control strategies

The virus can be prevented by avoiding direct contact with infected bats, pigs, and other animals. The infected patients should be strictly isolated to prevent human-to-human transmission. Some suggested measures include cleaning and disinfecting pig/animal farms, culling sick animals, limiting or prohibiting the transportation of animals from contaminated farms to other locations, and developing an animal health surveillance system. Additionally, the spread can be stopped by employing protective coverings (such as bamboo sap kilts) to keep bats away from sap-collecting sites and by wearing gloves and other protective gear when working with sick animals [51].

Limiting fruit collection, deforestation, and tourism in green spaces, as well as taking steps to combat climate change, can help reduce virus spillover into the environment. Strong measures against deceptive messaging, appropriate observance of preventive measures with reasonable understanding, and high alarms issued in impacted areas through public meetings, gatherings, and the conduct of large events can all help raise public awareness [51].

Healthcare personnel should strictly follow infection control protocols. Disinfectants such as sodium hypochlorite and autoclaving (heating to 100°C for 15 minutes) can be used to inactivate the virus in the hospital and laboratory settings. Healthcare providers should wear personal protective equipment (PPE), including a gown, gloves, eye protection, and an N95 respirator for protection when NiV is suspected. If NiV is confirmed, medical professionals should use PPE according to protocols for clinically unstable viral hemorrhagic fevers. The safety of hospitals and healthcare facilities can be ensured by establishing labs, regularly training medical personnel, isolating cases, tracking down and monitoring contacts, bolstering the surveillance system, and implementing containment measures [51].

The symptoms and prevention techniques should be thoroughly explained to persons at risk as part of community awareness campaigns. These include avoiding consuming fruits that have been bitten by bats, consuming raw date palm sap that has been tainted by bat excrement, and drinking the sap or molasses after boiling it; properly covering the vessels used to collect the sap; and being aware of and following hygienic procedures. In addition to preventing outbreaks, the aforementioned practices lower morbidity and death by facilitating early presentation and high suspicion of the infection [51].

The One Health approach acknowledges that the health of humans is inextricably linked to the health of animals and our common environment. A One Health strategy encourages collaboration among multiple professionals, including epidemiologists, laboratorians, physicians, and veterinarians, who function across human, animal, and environmental health to enhance the health of people and animals, including pets, livestock, and wildlife. The One Health approach can safeguard biodiversity, improve human and animal health, and reduce the spread of zoonotic illnesses. The strong relationship between globalization, urbanization, and the behavior of new viruses in the current era can be effectively addressed by this strategy [51].

**Figure 5:**
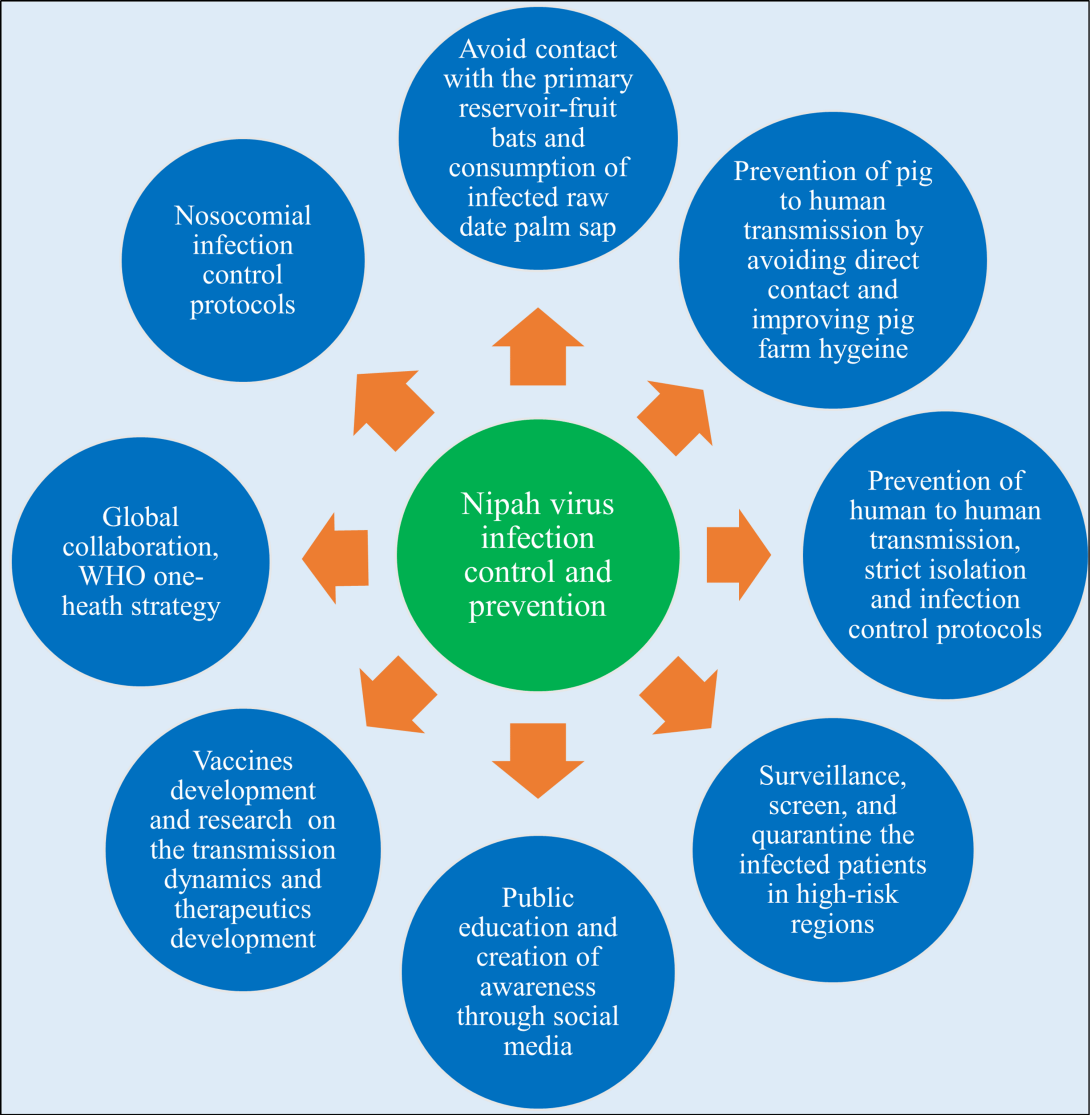
Nipah virus infection control and prevention strategies Image credit: Shreya Veggalam This image has been synthesized from references [51]. WHO: World Health Organization

Lastly, it is advised to increase funding for clinical trials for the development of vaccines and antiviral agents, as well as for epizootic research and stakeholder engagement, both domestically and internationally (Figure 5) [51].

NiV and the urgent need for vaccines

There isn't a licensed and authorized vaccination for NiV at the moment, although there are a lot of vaccine trials in progress. A NiV vaccine is desperately needed because of the infection's startlingly high CFR and frequent outbreaks in Bangladesh and India.

The rVSV∆G-EBOV GP/NiV G Vaccine 

The Vero cell cultures are used to create this live recombinant vesicular stomatitis virus (rVSV) viral vector, which co-expresses EBOV and NiV GPs. The EBOV GP facilitates cell entrance and fusion. It expresses the virus's NiV G protein, which is in charge of the virus's ability to adhere to the surface of the host cell. Antibodies against the G protein produced by vaccination prevent the virus from entering cells. According to early clinical research, the vaccine offers improved immunogenicity and has shown good protection in non-human primates. Currently, a phase-1 clinical trial (phase-1b, NCT06221813) is being conducted to assess the vaccine's immunogenicity and safety in healthy adult participants [70-72].

The ChAdOx1 Nipah B Vaccine

Clinical studies are being conducted on this vaccine, which was created by the Pandemic Sciences Institute at the University of Oxford. Using the same chimpanzee adenovirus vector platform as the Oxford/AstraZeneca SARS-CoV-2 vaccine, it is a recombinant adenoviral vector vaccine. It could be successfully administered and swiftly scaled up. Pre-clinical experiments are being conducted to evaluate the Jenner Institute's candidate vaccine based on AdOx1. It selectively targets the viral G GP, one of the key surface antigens on the NiV virion, to generate a robust immune response against the virus [73-75].

*The *messenger RNA (mRNA)*-1215 Vaccine*

This mRNA vaccine was created using lipid nanoparticles. The NiV-M strain's F and G proteins are among the viral GPs that it encodes. In September 2024, a phase-1 clinical investigation was concluded (NCT05398796). It has shown cross-reactivity with the HeV in mouse models, as well as immunogenicity and nAB responses against NiV-M and NiV-B [76].

HeV-sG-V Vaccine

It is a protein subunit vaccine, utilizing the soluble G GP of HeV (HeV-sG) formulated with aluminum hydroxide adjuvant. A phase 1 clinical trial (NCT04199169) was completed, and a phase 1b trial is ongoing. It offers protection against the Malaysian and the Bangladeshi strains. The notable outcomes in the phase-1 trial suggested that a single administration produced limited immunogenicity. The participants who received two doses of the vaccine have shown strong nABs against the virus [77].

Cluster of Differentiation (CD)-40.NiV (CD40.NiV)

**Figure 6:**
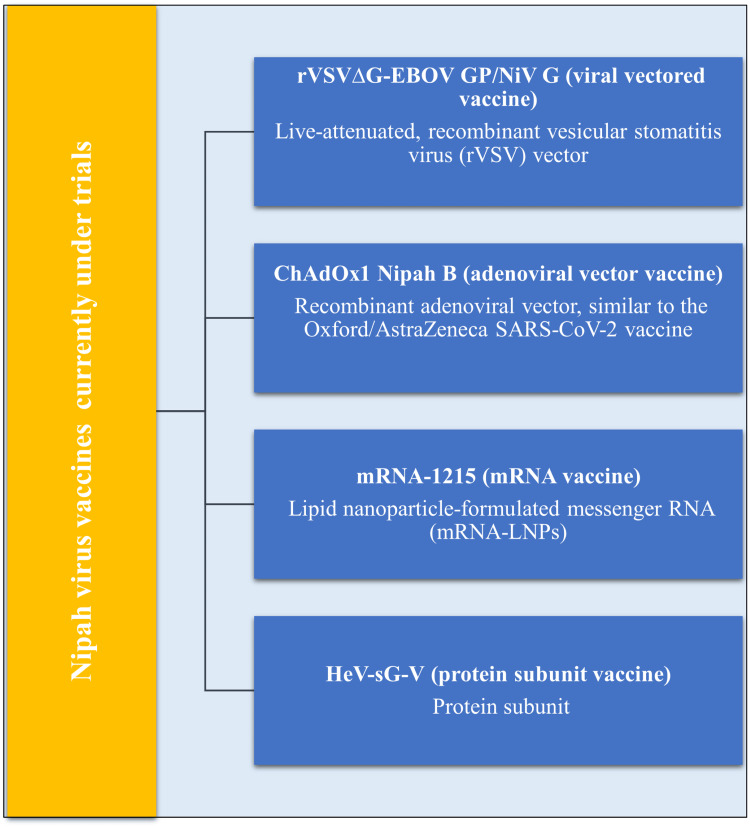
NiV candidate vaccines currently in human clinical trials Image credit: Shreya Veggalam NiV: nipah virus; rVSV: recombinant vesicular stomatitis virus; SARS-CoV-2: severe acute respiratory syndrome coronavirus 2; mRNA-LNPs: messenger ribonucleic acid lipid nanopaticles; HeV-sG-V: hendra virus soluble g protein vaccine

CD40.NiV is an anti-CD40 antibody to NiV proteins. It is a promising experimental vaccination for the NiV that targets antigen-presenting cells. It is being tested on animals and has shown promising results; all recipients of the vaccination have survived. Several strains of the NiV virus were neutralized by this vaccine (Figure 6) [78].

While more clinical testing is required to determine these candidate vaccines' safety and effectiveness in humans, they represent significant advancements toward containing and preventing NiV outbreaks. Immunoinformatics is a topic that bridges computer science and experimental immunology by using computational techniques and resources to comprehend and analyze immunological data. Some of the epitopes identified in the virus through immunoinformatic application include two bone marrow (B) cells, seven cytotoxic T lymphocytes (CTL), and seven helper T lymphocytes (HTL) epitopes that could enable the development of effective vaccines against NiV [79].

Critical insights into the mechanics of viral entry, host-cell fusion, and escape from the immune system have been gained from the biological structure of the NiV G and F GPs, laying the groundwork for the creation of vaccines and targeted treatments. Key epitopes and domains, like the G head domain, that are essential for nAB reactions have been identified by high-resolution structural investigations that have clarified the structure-dependent dynamics of these GPs. These findings have influenced the development of new vaccine candidates that seek to improve immunogenicity and provide broad-spectrum defense against NiV and related henipaviruses. Additionally, the discovery of weak spots on the G and F GPs has made it possible to produce mAbs, offering encouraging approaches for post-exposure treatments. Progress toward promising vaccine candidates has been further hastened by developments in protein engineering, such as enhancing the stability and scalability of antigen synthesis. Fighting new infections, particularly NiV with broad species tropism, will require combining structural biology with cutting-edge vaccination technology and therapeutic methods as the possibility of zoonotic spillover events increases. Our understanding of the biology of henipavirus will be strengthened by sustained and concerted international research into the structures and activities of NiV GPs. This may open the door to successful therapies that will stop future outbreaks and safeguard world health [80, 81].

Future perspectives

Recent conjectures on the transmission dynamics of NiV have shown that respiratory transmission may be a plausible mechanism in the majority of secondary, human-to-human transmissions. Inhaling droplets or aerosolized particles from mouth secretions and other bodily fluids could spread the virus. Aerosols or droplets that can be breathed can also be formed by blood and other bodily fluids. Ingestion, inhalation, or finger inoculation into the mouth, nose, or eyes could all result in transmission via fomites [39, 82, 83]. Given that NiV infection is characterized by a potential pandemic danger and is currently restricted to a narrow but lethal belt, other nations' attention is crucial. It has frequently been noted that NiV is a virus that has not received enough attention despite its significant harm potential [84]. 

Because NiV persists in reservoirs in nature and has a propensity to mutate into new varieties that could impact a wider spectrum of bat varieties and intermediate hosts, its eradication is still not feasible. This is made more difficult by climate change, which may accelerate viral development and raise the possibility of human transmission. Early diagnosis and control of human-to-human transmission are crucial because human illnesses put the virus under evolutionary strain. It is crucial to address the modifiable causes of NiV spillovers, and a critical assessment of health emergencies can improve subsequent tactics. It may be advisable for nations in eastern Africa, northern Australia, and southern and eastern Asia to evaluate their risk and put evidence-based, risk-informed preventative and control strategies into place. These should include periodical serosurveys of intermediate hosts, continuous tracking of bat colonies for NiV RNA and antibodies, and audits of deaths in at-risk human communities, as well as assessment for AES. More research is required to determine and change human behaviors that influence bat interactions and dissemination, such as eating fruits bitten by bats or date palm sap. Furthermore, in regions where transmission is active, other host intermediaries should be looked into, and sentinel animals such as horses and pigs should be subjected to repeated serosurveys. Early outbreak detection and control will be facilitated by implementing point-of-care diagnostic tests and bolstering AES surveillance. Strict infection control procedures, such as specialized isolation facilities and cutting-edge ventilation systems, must be implemented by hospitals in endemic locations. Research expenditures for antivirals, mAbs, and novel vaccines are essential. The assessment of pandemic potential will be aided by ongoing surveillance of NiV genotypes and clinical symptoms. It is important to keep an eye on survivors in case the virus reactivates. Creating resilient methods against NiV and other high-threat diseases will require a holistic one-health strategy that integrates ecological upheaval, changes in the climate, and human-animal interactions [85].

## Conclusions

Public health is seriously threatened by the NiV, especially in areas like India, where zoonotic transmission instances have been reported and where there is a risk of serious outbreaks. Proactively monitoring and managing this risk is crucial. Recent outbreaks, particularly in Kerala, have shown how urgently effective public health actions and robust surveillance systems are needed to control and lessen the risks associated with the NiV. The biological interactions between fruit bats and human activity highlight the necessity of implementing coordinated methods to address habitat degradation and instances of zoonotic spillover. Developing targeted therapies and workable therapeutic alternatives requires an understanding of the virus's biology and immune evasion mechanisms. To stop the outbreaks, international cooperation is crucial, along with improved surveillance techniques and research on treatments and vaccines. Since there is no specific therapy, preventing future epidemics requires early discovery, isolation of afflicted people, raising community awareness, and implementing stringent infection control procedures. Even in the absence of approved vaccines or targeted therapies, ongoing research into therapeutic medications and prospective vaccines may make future pandemic preparedness conceivable. Public health initiatives must be given top priority in order to lessen the impact of NiV outbreaks since they will familiarize people with not consuming raw date palm sap, which may contain the virus. This calls for an all-encompassing approach that unifies clinical, community-based, and environmental initiatives under a one health umbrella. Additional research on the pathophysiology, immunological response, and dynamics of NiV transmission, with reference to the emerging variants, is required in order to better comprehend and address this emerging viral threat.
